# Corneal absorption spectra in the deep UV range

**DOI:** 10.1117/1.JBO.27.2.025004

**Published:** 2022-02-26

**Authors:** Dominik Inniger, Alessio Poretti, Manuel Ryser, Christoph Meier, Christian Rathjen, Thomas Feurer

**Affiliations:** a University of Bern, Graduate School for Cellular and Biomedical Sciences, Bern, Switzerland; b Bern University of Applied Science, Engineering and Information Technology, HuCE-optoLab, Biel, Switzerland; c University of Bern, Institute for Applied Physics, Bern, Switzerland; d Ziemer Ophthalmic Systems AG, Port, Switzerland

**Keywords:** absorption, ultraviolet, ellipsometry, refractive index, ophthalmology

## Abstract

**Significance:**

Refractive surgery in ophthalmology uses pulsed lasers at 193, 210, or 213 nm. The reason is that most molecular constituents of cornea absorb strongly in this wavelength range. Precise refractive surgery via ablation requires an accurate knowledge of the absorption coefficient at the relevant wavelengths. Yet, the absorption coefficients of corneal tissue reported in literature vary by almost an order of magnitude; moreover, they were measured mostly at the wavelengths mentioned earlier.

**Aim:**

By measuring the corneal absorption coefficient of intact eyeballs stored at different environmental conditions, prepared by following different procedures, and as a function of postmortem time, we determine the absorption coefficient for the entire wavelength range between 185 and 250 nm for as close as possible to *in-vivo* conditions.

**Approach:**

We use a specially designed UV ellipsometer to measure refractive index and absorption coefficient. Specifically, we investigate the temporal evolution of refractive index and absorption coefficient after enucleation of the eyeballs under different environmental conditions and preparation procedures.

**Results:**

Our measurements provide accurate values for refractive index as well as absorption coefficient of cornea in the wavelength range between 185 and 250 nm. We find that the absorption coefficient decreases with time and that neither storage conditions nor preparation procedures but a continuous degeneration of the cornea is responsible for the observed time evolution. We use the measured time evolution to extrapolate refractive index and absorption coefficient to *in-vivo* conditions.

**Conclusion:**

Our measurements of the close to *in-vivo* absorption coefficient of cornea between 185 and 250 nm allow for a better understanding and modeling of refractive cornea surgery, also at other than the three commonly used wavelengths. In the future, this may be relevant when new pulsed laser sources with other wavelengths become available.

## 1 Context

For ophthalmologic applications, specifically for refractive surgery, the wavelength range between 190 and 220 nm has been identified as particularly suitable, because of the high absorption coefficient in corneal tissue in this part of the spectrum.^1^ The most commonly used pulsed laser sources are the ArF excimer laser with a wavelength of 193 nm^2^ and upconverted solid-state lasers at 210 and 213 nm.^3^ Studies on corneal ablation with these lasers have shown a similar degree of precision and tissue damage.^4^^–^^6^ Moreover, no significant difference in corneal mutagenesis was reported.^4^^,^^7^^,^^8^ The main difference is the two- to threefold higher absorption coefficient at 193 nm and, as a consequence, the lower threshold fluence for ablation.^9^^–^^11^ Corneal ablation experiments were also performed with KrCl and KrF excimer lasers at 222 and 248 nm, respectively, but the severe thermal effects on the surrounding tissue^12^ made these lasers unsuitable for refractive surgery.

Due to their fundamental importance for refractive surgery, a number of research groups have investigated corneal absorption coefficients. Interestingly, the reported values vary quite dramatically, for instance, at 193 nm literature values range from 2410 to $39,900\;{\mathrm{cm}}^{-1}$.^13^ A similar observation can be made for the absorption coefficient at 213 nm.^1^^,^^14^ The first measurement of the corneal absorption coefficient was in transmission mode using very thin slices to accommodate for the expected high absorption coefficients. The authors used a microtome to cut 32  μm (20  μm) thick slices from frozen dissected corneas^15^ (eyeballs^1^). Surprisingly, the results indicated an absorption coefficient as low as $2700\;{\mathrm{cm}}^{-1}$ ($2410\;{\mathrm{cm}}^{-1}$) at 193 nm, which is an order of magnitude lower than the $∼20,000\;{\mathrm{cm}}^{-1}$ estimated from the peptide bonds of the primary chromophores.^14^ Several subsequent indirect measurements, for instance ablation experiments,^16^ measurements on dissolved collagen,^13^ or interferometric photothermal spectroscopy^17^ suggested that the absorption coefficients should indeed be at least an order of magnitude higher. At 193 nm, they extracted values as high as 20,370, 16,000, and $19,000\;{\mathrm{cm}}^{-1}$, respectively. From these results, it became evident that reflection-type experiments could shed more light on the discrepancy. Reflection-type measurement do not rely on thin slices, because we may use intact eyeballs, are minimally invasive, and reduce dehydration of the cornea. Pettit and Ediger reported the first reflection measurements.^14^ They determined the reflection coefficient of a glass–cornea interface for different angles of incidence and extracted absorption coefficients of 39,900 and $21,400\;{\mathrm{cm}}^{-1}$ at 193 and 213 nm, respectively. Despite all advantages, reflection-type measurements can still yield fluctuating results, because sample storage conditions prior to the measurement or sample preparation can vary. For instance, samples have to be transported to the lab and hence are exposed to different ambient conditions (temperature, humidity, etc.) for up to several hours. The epithelium must be removed and superficial layers of water or physiological saline solutions are sometimes used to prevent degeneration of the cornea. For instance, at 193 nm, it has been shown that the ablation depth depends on the corneal hydration state^18^ and that saline solutions on the corneal surface may even act as a masking agent and hence may be used to treat corneal irregularities.^19^

The current state-of-the-art has motivated us to measure the corneal absorption coefficient in the range between 185 to 250 nm with a specially designed UV ellipsometer. We laid out the measurement protocols to assess the influence of storage conditions prior to the actual measurement as well as preparation procedures to, hopefully, extrapolate the absorption coefficient to *in-vivo* conditions. On the other hand, we aimed to cover the entire wavelength range between 185 and 250 nm to provide the absorption coefficient also for other than the three standard wavelengths. In the future, this may be relevant when new efficient pulsed laser sources with wavelengths different from the three standard sources at 193, 210, and 213 nm become available.

However, under ablation conditions additional dynamic effects may arise which modify light absorption. For instance, it has been reported that during ablative ArF laser irradiation light transmission decreases,^20^ tissue scattering properties change,^21^^,^^22^ and water absorption may increase by several orders of magnitude.^23^ Moreover, the ablation plume or plasma formation may decrease the fluence incident on the cornea.^24^ This effect is known to also depend on repetition rate and spot size. Hence, the small signal absorption coefficient is one of several but a fundamental ingredient to understand, model, and optimize refractive surgery.

We organized the paper as follows. We first outline the experimental setup, sample storage conditions, and preparation procedures. Then, we present the measurements of the absorption coefficient, compare the results to previous measurements, and discuss the influence of different environmental parameters. We use the results to extrapolate the absorption coefficient of cornea to *in-vivo* conditions.

## 2 Experiment

Ellipsometry is a standard technology to measure complex refractive indices or thin film thicknesses.^25^

**Fig. 1:**
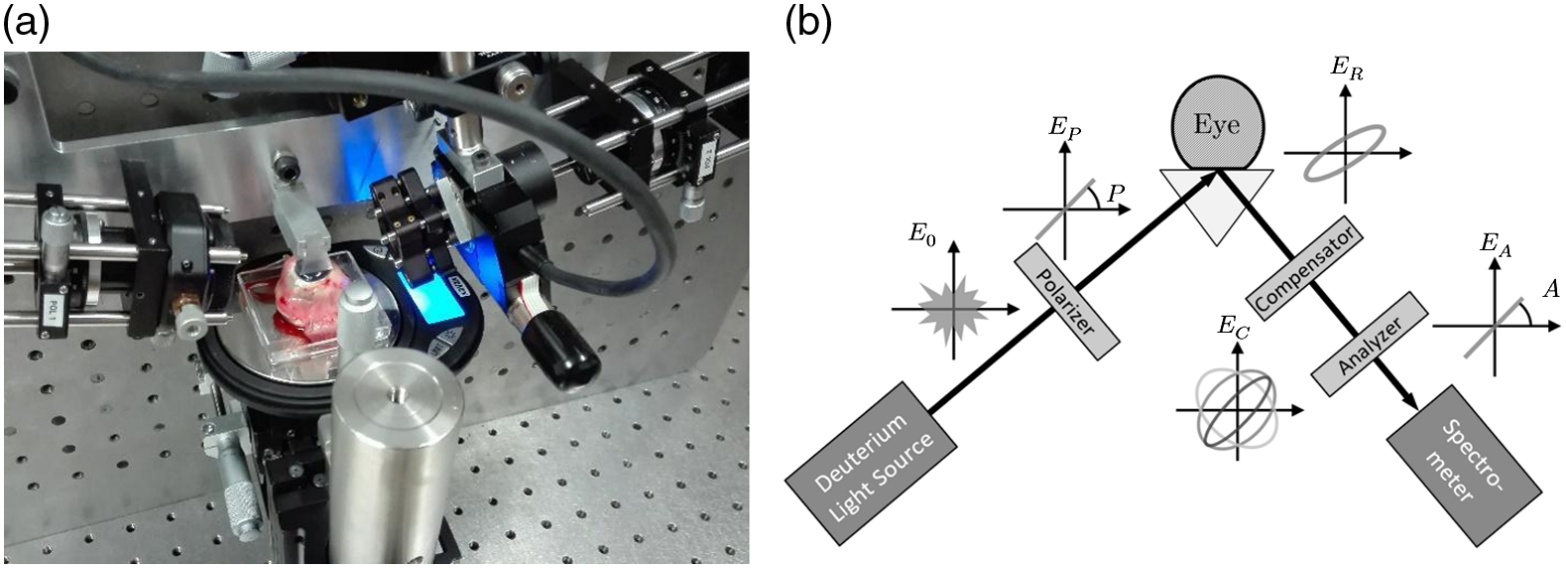
(a) Photographic image and (b) schematic of the RCE setup. A deuterium lamp coupled to a multimode fiber with collimator lens produces a broadband, nearly plane wave illumination. A downstream polarizer sets its polarization state. The light reflected from the sample is analyzed by a rotating compensator, which is followed by an analyzer and a UV spectrometer. The eyeballs are mounted on the base of an inverted UV prism.

In ellipsometry, a linearly polarized plane wave illuminates a sample so the incident light has an s- as well as a p-polarized electric field component with respect to the sample surface. The self-referencing detection scheme measures the difference between the complex-valued reflection coefficients for s- and p-polarized light, from which the optical properties of interest are extracted. Ellipsometry comes in different variants and the best-suited setup for our purpose is the “rotating compensator ellipsometry” (RCE), see Table 4.3 in Ref. 25. Figure 1 shows a photographic image and a schematic of the RCE setup. Knowing that the cornea’s index of refraction is of order 1.5 in the wavelength range of interest,^14^ we set the ellipsometer angle to 75 deg. The emitted broadband UV radiation from a 30 W deuterium lamp (BWTEK, BDS130A) is coupled in a 200 mm long solarization-resistant multimode patch fiber (Thorlabs FG200AEA) and collimated by a ${\mathrm{CaF}}_{2}$ lens (Thorlabs LA5315) to produce a broadband, nearly plane-wave illumination. A UV polarizer (Bernhard Halle Nachfl., PUM 1.08) with an azimuth angle of 45 deg defines the polarization state relative to the sample surface and an aperture after the polarizer limits the beam diameter to 2 mm. The sample holder is an inverted UV prism (Bernhard Halle Nachfl., Heraeus Suprasil 1, angles 30/75/75 deg, base size 14  mm×8  mm, and surface quality P4). A prism angle of 75 deg allows the incident and the reflected light to enter and to exit the prism normal to its side surfaces. After exiting the prism, the beam passes the compensator (Bernhard Halle Nachfl., RZM 4.10), which is mounted in a motorized rotation state (PI, RS-40 DC) and the analyzer (same type as polarizer). To maximize the dynamic range, the azimuth angle of the analyzer is set to −45  deg. The transmitted polarization component is focused (with the same lens as used for collimation) to a second 200 mm long multimode patch fiber (Thorlabs, FG105ACA), which is connected to the UV spectrometer (Ocean Insight, Maya2000 Pro-Deep-UV). The antireflection coatings of all optical elements as well as the operational wavelengths of polarizer, compensator, analyzer, and spectrometer allowed the ellipsometer to cover the wavelength range between 185 and 250 nm. The entire ellipsometer resided in a flow-box at room temperature.

For all measurements reported hereafter, we used pig eyes, which were enucleated within 3 min after death. Immediately after enucleation, the eyeballs were stored in a sealable container at a temperature of 4°C either on paper soaked with a 0.9% NaCl water solution or fully immersed in 0.9% NaCl water solution. Just before the measurement, the epithelium was removed with a hockey epithelium remover. Then, the eyeballs were mounted on a xyz translation stage and pressed against the prism base by moving only the z axis (perpendicular to the prism base) to avert shear forces. Moreover, we carefully prevented air entrapment and bubble formation at the interface. The time span between removing the eyeball from the cooled container to mounting it was typically 2 min. The mount was equipped with a thermocouple (0.1 K resolution) and a scale (0.1 g resolution) to monitor temperature and contact force before, during, and after the measurements. The initial contact force was below 100 mN. Then, we let the system relax for 5 to 15 min (stabilization time) while continuously monitoring the spectrally resolved ellipsometer signal and the measurement was started when the signal change was below 5‰ per minute. At this point, the contact force was below 70 mN and the temperature at the bottom of the eyeball was between 14 to 17°C.

Since the polished prism surface (P4, ISO 10110-8) defines the interface and cornea is highly absorbing, we assume a perfectly flat sample with infinite thickness to extract the cornea’s pseudodielectric function. Note that the microscopic structure of the stroma consists of collagen fibril bundles called lamellae, which per se are birefringent.^26^ However, these bundles are randomly oriented within the central part of the cornea,^27^ and we assume a zero net birefringence when probing an area of $∼12\;{\mathrm{mm}}^{2}$. For a single measurement, we rotated the compensator angle C by 180 deg in steps of 10 deg. At every angle twelve spectra, each with an integration time of 80 ms, were recorded and averaged. The total time for an entire scan was 2.5 min and we define the postmortem time as the time elapsed between enucleation and end of measurement. For the RCE configuration, the signal as a function of the compensator angle C is^28^^,^^29^

$$I_{D}(C)=a_{0}+a_{2c}\;\cos2C+a_{2s}\;\sin2C+a_{4c}\;\cos4C+a_{4s}\;\sin4C.$$ (1)

The wavelength-dependent Fourier coefficients $a_{i}$ are defined via

$$a_{0}=\frac{1}{2}I_{0}(1+\cos\;\delta_{c})(\sin\;2A\sin\;2\xi \cos\Delta -\cos\;2A\cos\;2\xi )+I_{0}\;a_{2c}=-I_{0}\sin\;2A\sin\;2\xi \sin\Delta \sin\;\delta_{c}\;a_{2s}=I_{0}\cos\;2A\sin\;2\xi \sin\Delta \sin\;\delta_{c}\;a_{4c}=\frac{1}{2}I_{0}(\cos\;\delta_{c}-1)(\sin2A\sin\;2\xi \cos\Delta +\cos\;2A\cos\;2\xi )\;a_{4s}=-\frac{1}{2}I_{0}(\cos\;\delta_{c}-1)(\cos\;2A\sin\;2\xi \cos\Delta -\sin\;2A\cos\;2\xi ),$$ (2)

with the compensator phase retardance $\delta_{c}$, the analyzer angle A, the amplitude ratio of p- and s-polarized light after reflection from the sample ξ=atan(tan Ψ/tan P), the polarizer angle P, and the three sample parameters $I_{0}$, Ψ, and Δ. Fitting Eq. (1) for every wavelength to the measured data reveals the ellipsometry parameters Ψ and Δ from which we extract the wavelength-dependent pseudodielectric function^25^

$$\varepsilon_{t}(\Psi ,\Delta )=\varepsilon_{i}\;\tan\;\theta_{i}[1+\tan\;\theta_{i}(\frac{1-\tan\;\Psi e^{-i\Delta }}{1+\tan\;\Psi e^{-i\Delta }}{)}^{2}],$$ (3)

with the dielectric function of the prism material $\varepsilon_{i}$ and the incident angle $\theta_{i}$. From the definition $n+ik=\sqrt{\varepsilon_{t}}$, the real and the imaginary parts of the refraction index are calculated. The absorption coefficient is obtained via

$$\alpha =\frac{4\pi }{\lambda }k.$$ (4)

By taking into account the alignment precision of all relevant optical elements, we find that the absolute error decreases from $\pm 1400\;{\mathrm{cm}}^{-1}$ at 185 to $\pm 600\;{\mathrm{cm}}^{-1}$ at 200 nm and remains constant at $\pm 600\;{\mathrm{cm}}^{-1}$ above.

## 3 Results and Discussion

### 3.1 Ellipsometer Calibration

To calibrate the ellipsometer system, we performed two measurements, one at a prism–air and the other at a prism–distilled water interface, with the goal to correct for imperfect optical elements.

**Fig. 2:**
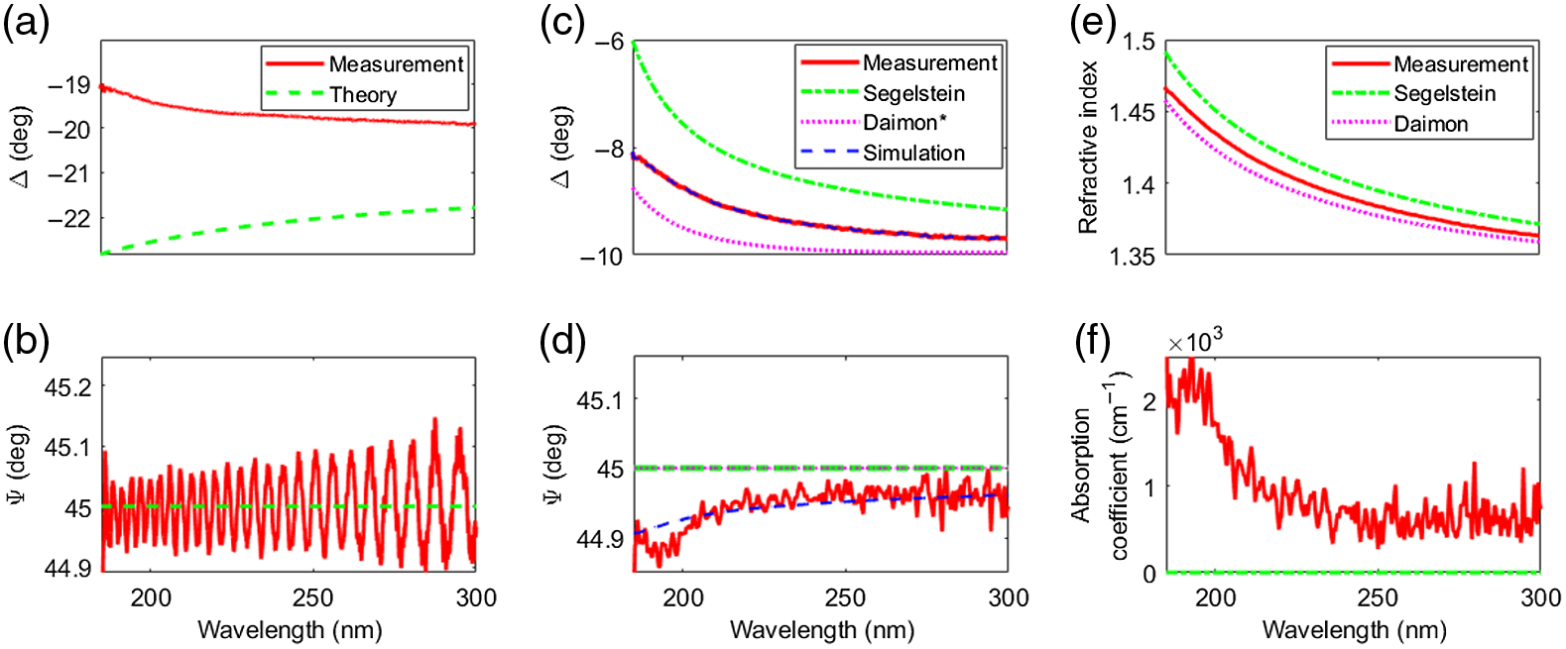
Calibration of the ellipsometer. Measured (solid red curve) and theoretical (dashed green curve) (a) phase difference Δ and (b) amplitude ratio Ψ for a prism–air interface. Measured (solid red curve) and theoretical (dashed green curve: Segelstein;^30^ dotted pink curve: Diamon^31^ assuming zero absorption) (c) phase difference Δ and (d) amplitude ratio Ψ for a prism–water interface. The simulations (dashed blue curve) take into account a rotation by 11 deg of the prism birefringence with respect to the beam coordinate system. (e) Measured refractive index and (f) absorption coefficient compared with literature values.^30^^,^^31^

Figure 2 shows theoretical (dashed green curve) and measured (solid red curve) phase difference Δ (a) and amplitude ratio Ψ (b) as a function of wavelength for the prism-air interface. For Δ, we find a deviation from the expected values between 2 and 4 deg, which we attribute to a stress induced birefringence of the prism. This has been confirmed by exerting additional pressure on the prism. The average measurement of Ψ agrees well with the expected value of 45 deg and the oscillations with an amplitude of 0.1 deg result from an imperfect compensator. Both deviations $\delta \Delta_{\mathrm{air}}\;$and $\delta \Psi_{\mathrm{air}}$ are used to correct further measurements.

Next, we perform a measurement on distilled water with the aim to determine whether the birefringence of the prism is rotated with respect to the beam coordinate system. We use water because its refractive index is closer to that of cornea. The measured ellipsometry parameters Δ and Ψ (solid red curves) are shown in Figs. 2(c) and 2(d) and the resulting index of refraction and absorption coefficient in Figs. 2(e) and 2(f), respectively. While the measured refractive index is between the two literature values, the absorption coefficient is significantly higher than the literature values especially below 225 nm. If we rotate the coordinate system of the prism birefringence by 11 deg, we find perfect agreement between the measured and the simulated results. Note that the rotation affects only the amplitude ratio Ψ, and hence the absorption coefficient, but does not alter the phase difference Δ. Hence, we find $\delta \Delta_{\mathrm{water}}=0$ and a nonzero $\delta \Psi_{\mathrm{water}}$. As a result of the calibration measurements, all subsequent measurements on pig eyes are corrected by ($\delta \Delta_{\mathrm{water}}+\delta \Delta_{\mathrm{air}}$) and ($\delta \Psi_{\mathrm{water}}+\delta \Psi_{\mathrm{air}}$) before the index of refraction and the absorption coefficient are calculated.

### 3.2 Cornea

**Fig. 3:**
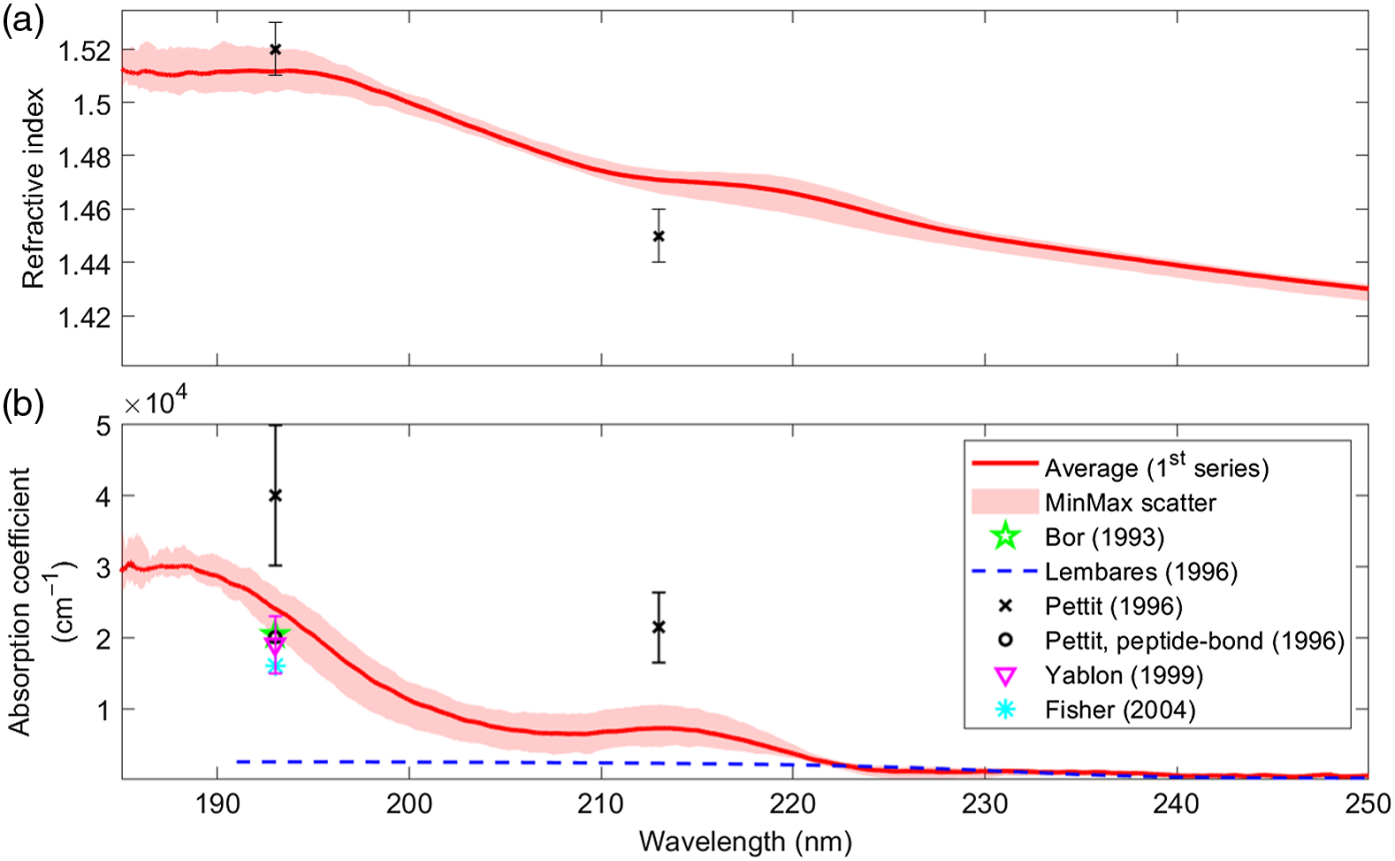
(a) Refractive index and (b) absorption coefficient of porcine cornea between 185 and 250 nm. The red curves represent the average values and the light red-shaded regions indicate the range between the minimum and maximum values. Literature values are included as indicated in the legend.

Figure 3 shows the real part of the refractive index and the absorption coefficient of porcine cornea as a function of wavelength between 185 and 250 nm. In detail, 10 eyeballs, fully immersed in 0.9% NaCl solution, were prepared one after the other as described above. The time span between two subsequent measurements was 45 min and all 10 eyeballs were measured between 2 and 9 h postmortem. While the solid red curves indicate mean values, the light red shaded regions indicate the range between the minimum and the maximum values. We would like to stress that the maximum curve corresponds tendentially to the first and the minimum curve to the last measurement, in other words, during the measurement time of 7 h, the refractive index and the absorption coefficient were slowly decreasing.

The real part of the refractive index shows a continuous increase toward shorter wavelengths. Below 195 nm, we find an almost constant value of 1.51. The refractive index in the entire wavelengths range is significantly higher than that of water and exhibits a slightly more complex behavior. The values published by Pettit et al.^14^ (eyes stored in a plastic bag on ice) for 193 and 213 nm deviate by 0.01 at 193 nm and −0.02 at 213 nm. The absorption coefficient also increases with decreasing wavelength but shows two peaks around 187 nm with a peak absorption coefficient of $30,000\;{\mathrm{cm}}^{-1}$ and around 213 nm with a peak absorption coefficient of $7200\;{\mathrm{cm}}^{-1}$. Between 204 and 216 nm, the absorption coefficient is roughly constant on a level of $7000\;{\mathrm{cm}}^{-1}$. For wavelengths larger than 225 nm, the absorption coefficient drops below $2000\;{\mathrm{cm}}^{-1}$, which agrees well with the transmission measurement of Lembares et al.;^1^ below 225 nm, however, we observe a significant deviation from these transmission measurements. A better agreement is found when comparing our data to the results of Pettit et al.,^14^ who reported $(39,900\pm 9800)\;{\mathrm{cm}}^{-1}$ at 193 nm and $(21,400\pm 4900)\;{\mathrm{cm}}^{-1}$ at 213 nm. The two values deviate from our results by 15,900 and $14,200\;{\mathrm{cm}}^{-1}$, respectively. At 193 nm, we find more literature values,^13^^,^^14^^,^^16^^,^^17^ but all of them are lower by 4000 to $8000\;{\mathrm{cm}}^{-1}$ when compared with our values. As discussed in Sec. 1, we believe that these deviations are mostly due to different sample preparation, measurement technique, or degree of sample degeneration. To further investigate these issues, we carried out additional measurements under different environmental conditions.

We started by investigating the influence of the contact force with which the eyeballs were pressed against the prism. When mounted, we apply a contact force of ∼100  mN, which relaxes to <70  mN after the stabilization time. When applying contact force in excess of 100 mN, we found a stress-induced birefringence in the prism that corrupts the measurements.

Next, we investigated sample degeneration possibly caused by UV radiation. We prepared two identical series of samples and recorded reference spectra for both. Then, we continuously irradiated one of them while shielding the other from the UV radiation. After the stabilization time, we again recorded spectra and found no evidence of UV-related sample degeneration.

The next four series of measurements were designed to better understand the influence of the hydration state of the corneal surface. Prior to the measurement, all eyeballs were stored in a sealable container at a temperature of 4°C. While the eyeballs of the first series were immersed in 0.9% NaCl water solution, the eyeballs of the second, third, and fourth series were stored on paper soaked with 0.9% NaCl water solution. We removed the epithelium of the first and second series and mounted and measured them immediately after. Similarly, the epithelium of the third series was removed but the stroma was moistened with a 0.9% NaCl water solution before mounting them. Finally, the eyeballs of the fourth series were treated as those of the second series, but they were stored about 25 h longer. The conditions are summarized in Table 1.

**Table 1:** Measurement series and corresponding measurement conditions.

Series	Storing condition	Preparation	# of eyeballs in series	Measurement time
1	Sealable container at 4°C, eyeballs immersed in 0.9% NaCl water solution	Measured immediately after epithelium removal	10	First eyeball: 2 h
				Last eyeball: 9 h
2	Sealable container at 4°C, eyeballs on paper soaked with 0.9% NaCl water solution	Measured immediately after epithelium removal	5	First eyeball: 5 h
				Last eyeball: 11 h
3	Sealable container at 4°C, eyeballs on paper soaked with 0.9% NaCl water solution	After epithelium removal, stroma was moistened with 0.9% NaCl water solution	4	First eyeball: 4 h
				Last eyeball: 10 h
4	Sealable container at 4°C, eyeballs on paper soaked with 0.9% NaCl water solution	Measured immediately after epithelium removal	5	First eyeball: 30 h
				Last eyeball: 35 h

**Fig. 4:**
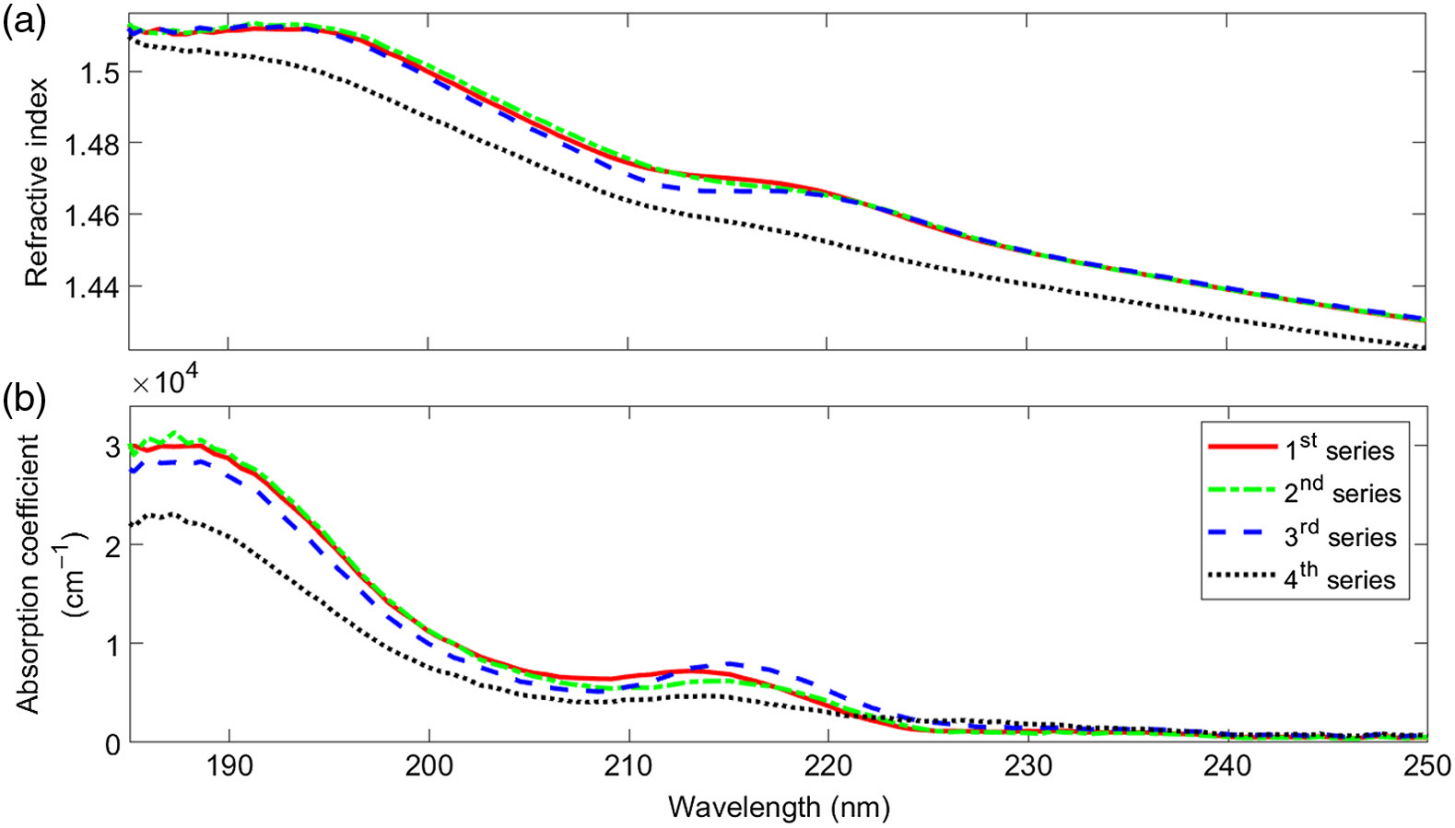
(a) Refractive index and (b) absorption coefficient of porcine corneas between 185 and 250 nm for the four different series. For details, see Table 1 and text.

Figure 4 compares the refractive indices as well as the absorption coefficients of the four series. The results suggest that neither the storage conditions (immersed in 0.9% NaCl water solution or stored on paper soaked with a 0.9% NaCl water solution) nor moisturizing the corneal surface with a 0.9% NaCl water solution prior to mounting has any major influence on the results. The only significant difference we observe when eyeballs are stored for longer times. In this case, we find a decrease of the refractive index as well as of the absorption coefficient for the most part of the spectrum.

**Fig. 5:**
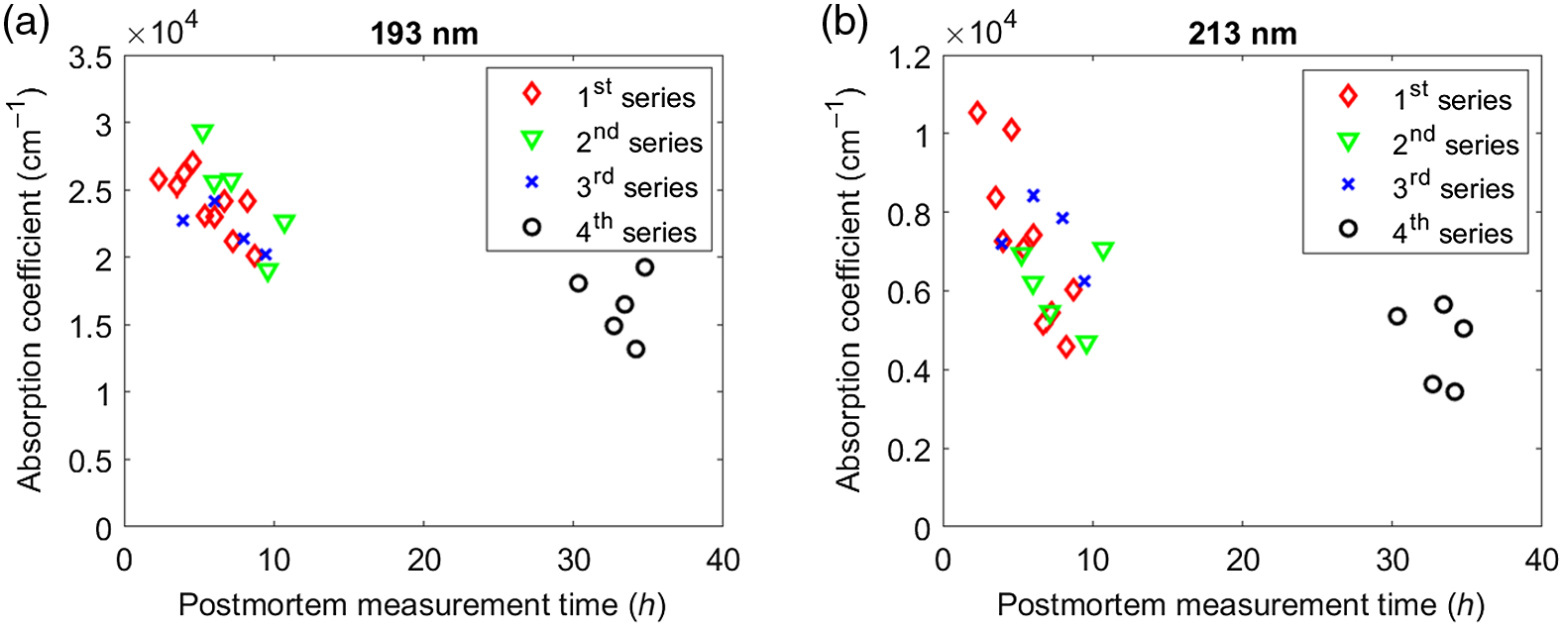
Absorption coefficient at different postmortem measurement times (a) 193 nm and (b) 213 nm.

These findings led us to the conclusion that a continuous slow degeneration of the cornea is the main reason for the decreasing absorption coefficient. This becomes even more obvious when we plot the absorption coefficient at 193 and 213 nm as a function of postmortem time for all series of eyeballs investigated above. Figures 5(a) and 5(b) show that, irrespective of storage condition or preparation procedure, the absorption coefficient always decreases with time, and the effect is somewhat more pronounced at 213 nm than at 193 nm. Similar observations are also reported elsewhere in the literature. For instance, it has been found that the thickness of the cornea changes within 30 min postmortem^32^ and that the corneal hydration^33^ and the collagen diameter of the corneal matrix^34^ increase with postmortem time. Because collagen fibers dominate the corneal UV absorption below 220 nm, we believe that the decreasing absorption coefficient is related to an increased hydration level or the associated structural changes. Needless to say, that the observed degeneration is irrelevant in refractive surgery, since the procedure takes place within a few minutes and the endothelium is not affected (responsible for the active dehydration process).^26^

**Fig. 6:**
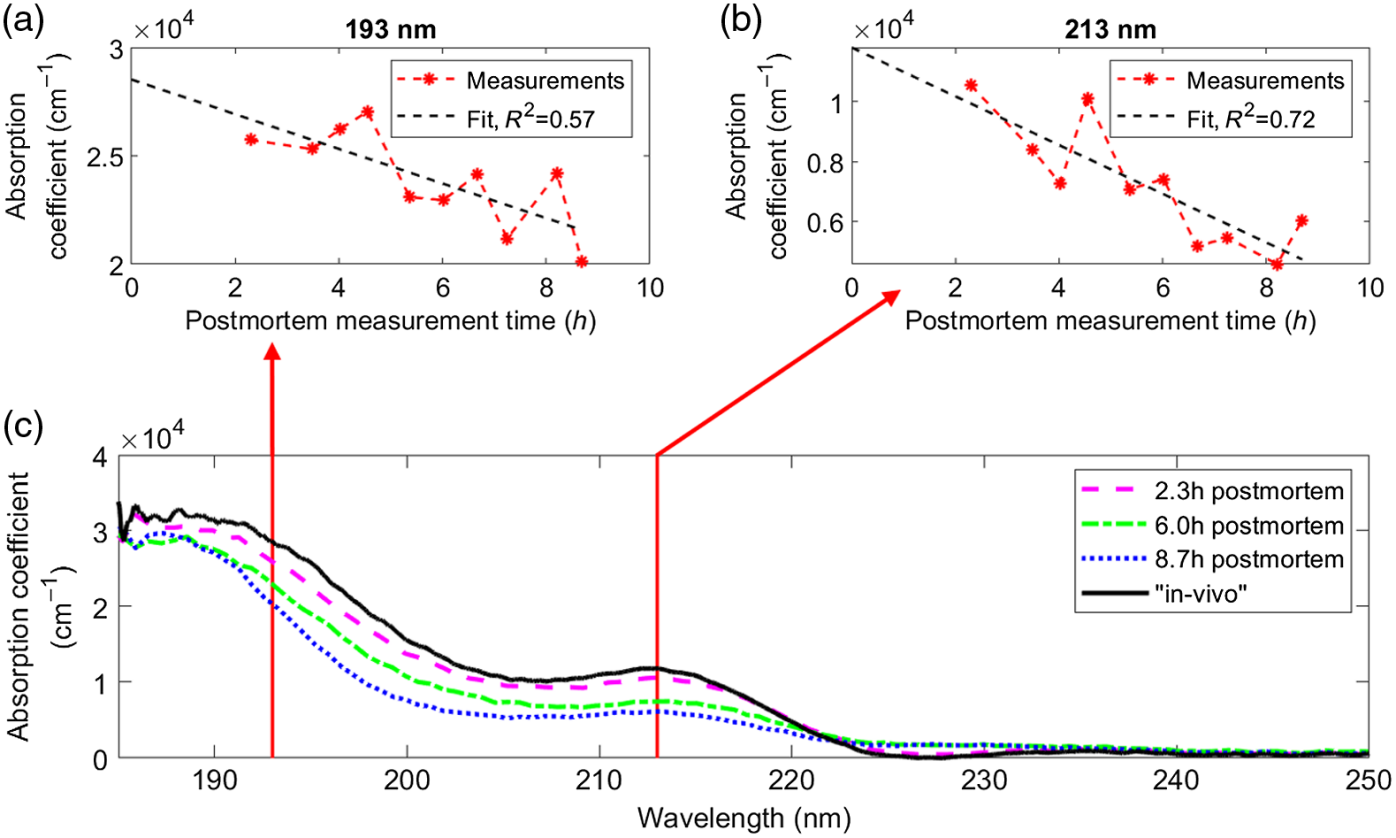
Absorption coefficient of the first series at (a) 193 nm and (b) 213 nm as a function of postmortem time. Applying a linear regression (dashed black lines) to the measured data (red stars) allows us to extrapolate the absorption coefficient to the time of death. (c) Absorption coefficient measured 2.3 h (pink curve), 6 h (green curve), and 8.7 h (blue curve) postmortem and the absorption coefficient extrapolated to the time of death (black curve). The two vertical red lines indicate the two wavelengths shown in (a) and (b).

Zooming in on the first 9 h postmortem of the first series strongly suggests that the absorption coefficient decreases more or less linearly within this time span. This allows us to extrapolate the wavelength-dependent absorption coefficient to time zero, i.e., to the time of death. Exemplary, we show the linear fit in Figs. 6(a) and 6(b) for the two important wavelengths of 193 and 213 nm.

Figure 6(c) shows the absorption coefficient measured 2.3 h (pink curve), 6 h (green curve), and 8.7 h (blue curve) postmortem and the absorption coefficient extrapolated to time zero, i.e., the time of death (black curve). The result of the fitting procedure is the “*in-vivo*” corneal absorption coefficient between 185 and 250 nm.

## 4 Conclusion

A specially designed UV ellipsometer was used to determine the absorption coefficient of porcine cornea in the wavelength range from 185 to 250 nm. Measurements up to 35 h postmortem reveal that the absorption coefficient continuously decreases with time for the most part of the spectrum. The observed decrease does not correlate with the UV irradiation dose, the applied force, nor the storage condition, but relates to a continuous slow degeneration of the cornea. Using the measured time series allows for extrapolating the absorption coefficient to the time of death and to report, for the first time, the “*in-vivo*” absorption coefficient in the wavelength range between 185 and 250 nm.

## Code, Data, and Materials Availability

Theory, original data, and data processing supporting the plots in this paper are available via DOI number 10.48350/161507.
